# 

**Published:** 1873-07

**Authors:** 


### TABLE OF CONTENTS.

### ORIGINAL COMMUNICATIONS.

ERGOT AS A REMEDIAL AGENT IN EPIDEMIC CHOLERA. By G. E. SURSSDORFF, M.D.........183

HERPES ZOSTER—OPHTHALMICUS. By A. W. CALHOUN, M.D.........188

PULMONARY TUBERCULOSIS. By A. W. GRIGGS, M.D.........191

CASE OF COLICA PICTONUM. By JOHN P. RALLS, M.D.........196

ATLANTA ACADEMY OF MEDICINE. JAS. P. BAIRD, M.D., Reporter.........197

ŒDEMA OF SCROTUM AND PENIS, ETC. By W. S. ARMSTRONG, M.D.........200

ABSTRACTS FROM GERMAN JOURNALS. By Ch. RAUSCHENBERG, M.D.........205

(Suggestions in Relation to the Treatment of Cholera.)

HYDROCHLORATE OF AMMONIA. By K. P. MOORE, M.D.........214

BLOOD-LETTING, A VALUABLE CURATIVE MEANS, ETC. By JAMES RODGERS, M.D.........220

REPORT OF ROBERT BATTEY, M.D., DELEGATE TO THE ALABAMA MEDICAL ASSOCIATION.....224

### EDITORIAL AND MISCELLANEOUS.

CONTRIBUTIONS TO THE MEDICAL PRESS.........228
SAVANNAH MEDICAL COLLEGE.........230
EDITORIAL CORRESPONDENCE.........231
BROMIDE OF POTASSIUM.........232
NORMAL ACTION OF THERAPEUTIC AGENTS.........232
TREATMENT OF TRISMUS AND TETANUS BY HYDRATE OF CHLORAL.........233
ATLANTA MEDICAL COLLEGE.........234
BIBLIOGRAPHICAL.........235
CARBOLIC ACID IN TREATMENT OF DYSENTERY.........236
CHOLERA.........237
HYOSCYAMINE IN SPASMODIC AND CONVULSIVE NEUROSIS, ETC.........240
BELLADONNA VERSUS OPIUM, AND OPIUM VERSUS BELLADONNA.........240

